# The STING HAQ haplotype and clinical non-penetrance in COPA syndrome

**DOI:** 10.1084/jem.20251470

**Published:** 2026-03-10

**Authors:** Clémence David, Tifenn Wauquier, Alix de Becdelièvre, Camille Louvrier, Maud Tusseau, Cécile Masson, Luis Seabra, Caroline Kannengiesser, Hayssam Al Arab, Ibrahima Ba, Mary Brennan, Alexandre Belot, Nadia Nathan, Hélène Maillard, Héloïse Reumaux, Jérémie Sellam, Jacques Cadranel, Yves Hatchuel, Laurence Weiss, Sébastien de Almeida, Cinthia Rames, Marie Wislez, Clémentine Vigier, Géraldine Labouret, Claire Kastner, François Provot, Julien Tarabeux, Elise Schaefer, Darragh Duffy, Vincent Bondet, Paul Bastard, Anne Puel, Jean-Laurent Casanova, Gillian I Rice, Brigitte Bader-Meunier, Yanick J Crow, Alice Lepelley, Marie-Louise Frémond

**Affiliations:** 1 Laboratory of Neurogenetics and Neuroinflammation, Imagine Institute, Inserm U1163, F-75015, Paris, France; 2 Department of Internal Medicine, Hôpital Bichat-Claude Bernard, Assistance Publique Hôpitaux de Paris, Université Paris Cité, F-75018, Paris, France; 3 Laboratory of Genetics, Henri Mondor University Hospital, APHP, University Paris Est Creteil, INSERM, IMRB, F-94000, Creteil, France; 4 Laboratory of Childhood Genetic Diseases, UMR_S933, Sorbonne University, INSERM, Armand Trousseau Hospital, F-75012, Paris, France; 5 Hospices Civils de Lyon, Department of Medical Genetics, University Hospital of Lyon, F-69002 Lyon, France; 6 Bioinformatics Core Facility, Université Paris Cité- Structure Fédérative de Recherche Necker, INSERM US24/ CNRS UMS3633, F-75015, Paris, France; 7 Service de Génétique, Hôpital Bichat, Assistance Publique Hôpitaux de Paris, Université Paris Cité, F-75018, Paris, France; 8 National Reference Centre for Inflammatory Rheumatism, Autoimmune Diseases and Systemic Interferonopathies in Children (RAISE), Paediatric Nephrology, Rheumatology, Dermatology Unit, Hospital of Mother and Child, Hospices Civils of Lyon, F-69677, Bron, France; 9 Paediatric & Adolescent Rheumatology, Royal Hospital for Children & Young People, Edinburgh, United Kingdom; 10 Paediatric Pulmonology Department and Reference Centre for Rare Lung Diseases RespiRare, INSERM UMR_S933 Laboratory of Childhood Genetic Diseases, Armand Trousseau Hospital, Sorbonne University and APHP, F-75012, Paris, France; 11 Department of Internal Medicine and Clinical Immunology, Referral Centre for Rare Systemic Autoimmune Diseases in the North of France, Northwest, Mediterranean and Guadeloupe (CeRAINOM), CHU de Lille, F-59000, Lille, France; 12 Paediatric Rheumatology Unit, University of Lille, Jeanne de Flandre Hospital, F-59800, Lille, France; 13 Department of Rheumatology, Saint-Antoine Hospital, APHP, Centre de Recherche Saint-Antoine (CRSA) Inserm UMRS_938, Sorbonne Université, F-75012, Paris, France; 14 Department of Pneumology and Thoracic Oncology, Tenon Hospital, Assistance Publique-Hôpitaux de Paris (AP-HP) Sorbonne Université, F-75020, Paris, France; 15 Department of General Paediatrics, Competence Centre for Inflammatory Rheumatism, Autoimmune Diseases and Systemic Interferonopathies in Children (RAISE) Antilles-Guyane, EpiCliV Research Unit, University of the French West Indies, Martinique University Hospital, F-97234, Fort-de France, France; 16 Pediatric Pulmonology, Strasbourg University Hospital, F-67000, Strasbourg, France; 17 Department of Internal Medicine, CHU de Toulouse, F-31000, Toulouse, France; 18 Paediatric Pneumology and Allergology Unit, CHU of Amiens, F-80000, Amiens, France; 19 Service de Pneumologie, Hôpital Cochin, AP-HP, Inserm U1138, Université Paris Cité, F-75014, Paris, France; 20 Paediatric Pneumology Unit, CHU of Rennes, F-35033, Rennes, France; 21 Paediatric Pulmonology Department, University Hospital for Children, F-31000, Toulouse, France; 22 Institut de Génétique Médicale d’Alsace, F-67000, Strasbourg, France; 23 Université de Lille, CHU Lille, Nephrology Department, F-59000, Lille, France; 24 Translational Immunology Unit, Institut Pasteur, Université Paris Cité, F-75015, Paris, France; 25 Laboratory of Human Genetics of Infectious Diseases, Necker Branch, INSERM U1163, Necker Hospital for Sick Children, Paris, France, EU. Paris Cité University, Imagine Institute, Paris, France; 26 St. Giles Laboratory of Human Genetics of Infectious Diseases, Rockefeller Branch, The Rockefeller University, New York, NY, United States of America; 27 Paediatric Haematology-Immunology and Rheumatology Unit, Necker Hospital, AP-HP, F-75015, Paris, France; 28 Howard Hughes Medical Institute, New York, NY, United States of America; 29 Division of Evolution Infection & Genomic Sciences, School of Biological Sciences, Faculty of Biology, Medicine and Health, University of Manchester, Manchester, United Kingdom; 30 Paediatric Haematology-Immunology and Rheumatology Unit, Necker Hospital, AP-HP, Université Paris Cité and Reference Centre for Inflammatory Rheumatism, Autoimmune Diseases and Systemic Interferonopathies in Children (RAISE), F-75015, Paris, France; 31 MRC Human Genetics Unit, Institute of Genetics and Cancer, University of Edinburgh, Edinburgh, United Kingdom

## Abstract

COPA syndrome is a rare monogenic autoinflammatory disease due to heterozygous mutations in *COPA*, encoding the coatomer subunit α. COPA syndrome demonstrates phenotypic overlap with SAVI (STING-associated vasculopathy with onset in infancy), the latter due to gain-of-function mutations in *STING1*. Indeed, STING activation is a key driver of the pathogenesis of COPA syndrome, and a recent report suggested that the presence of the common HAQ STING allele confers complete protection against the development of clinical disease in the context of pathogenic heterozygous mutations in *COPA*. Given the potential significance of this result for patient management, we investigated the STING HAQ haplotype status of a separate cohort of individuals segregating pathogenic mutations in *COPA*. In doing so, we ascertained five HAQ negative, clinically asymptomatic individuals aged 30, 39, 39, 42 and 43 years at last evaluation, and an HAQ positive male with kidney disease that we consider most likely attributable to the recurrent R233H mutation in COPA. Our findings challenge the suggestion that STING haplotype status is the sole determinant of clinical penetrance in COPA syndrome.

## Non-standard abbreviations

BAL: bronchoalveolar lavage
CT: computed tomography
IEIs: inborn errors of immunity
IFN-I: type I interferon
PFTs: pulmonary function tests

## Introduction

The field of autoinflammatory disorders has expanded considerably over the past 25 years. Alongside the discovery of novel genetic variants resulting in the inappropriate activation, or defective negative regulation, of inflammatory molecules, striking examples of clinical non-penetrance have been recorded, including among the inherited type I interferonopathies - autoinflammatory diseases characterized by constitutive activation of the type I interferon (IFN-I) pathway. For example, in a study of 74 individuals harbouring pathogenic heterozygous gain-of-function mutations in *IFIH1*, 13.5% (seven of whom were aged over 50 years) were clinically asymptomatic (Rice et al., 2020). More recently, asymptomatic individuals homozygous for the most common mutation observed in symptomatic patients with *RNASEH2B*-related Aicardi-Goutières syndrome were reported, thereby highlighting the possibility of clinical non-penetrance also in the context of autosomal recessive disease (Crow et al., 2023).

COPA syndrome is a type I interferonopathy caused by autosomal dominant mutations in *COPA* (Watkin et al., 2015). The pathogenesis of COPA syndrome is directly linked to STING function, as COPA mutations result in a defect of the retrograde transport of STING (Deng et al., 2020; Lepelley et al., 2020; Mukai et al., 2021; Steiner et al., 2022). Where COPA function is disturbed, STING - an innate immune protein essential to the induction of an IFN-I response following the sensing of cytosolic DNA - remains activated in the Golgi, thereby mediating chronic IFN-I signalling. The frequency of clinical non-penetrance among individuals heterozygous for bona fide pathogenic mutations in *COPA* is remarkably high, having been previously estimated at 15 - 20% (Lepelley et al., 2020; Simchoni et al., 2023, David et al., 2025). Noting both that STING haplotypes can influence the potency of downstream IFN signalling (Yi et al., 2013) and the pivotal role of STING in the pathogenesis of COPA syndrome, Simchoni et al. recently evaluated the potential effect of STING haplotypes in the clinical penetrance / non-penetrance of COPA syndrome (Simchoni et al., 2025). In doing so, they found the common STING HAQ haplotype, combining three non-synonymous substitutions i.e. R71H, G230A and R293Q, to be present in all nine clinically asymptomatic individuals heterozygous for a COPA mutation (either R233H, A239P, E241K, V242D or D243G) assessed in their cohort. Conversely, the STING HAQ haplotype was absent in all 26 individuals manifesting clinical disease that they tested. Experimentally, they further demonstrated that HAQ STING acts dominantly to dampen COPA-dependent STING activation. Given the potential importance of these data for clinical management, we decided to investigate the STING HAQ haplotype status of a separate cohort of individuals segregating pathogenic mutations in *COPA*.

## Results and discussion

**Figure 1:**
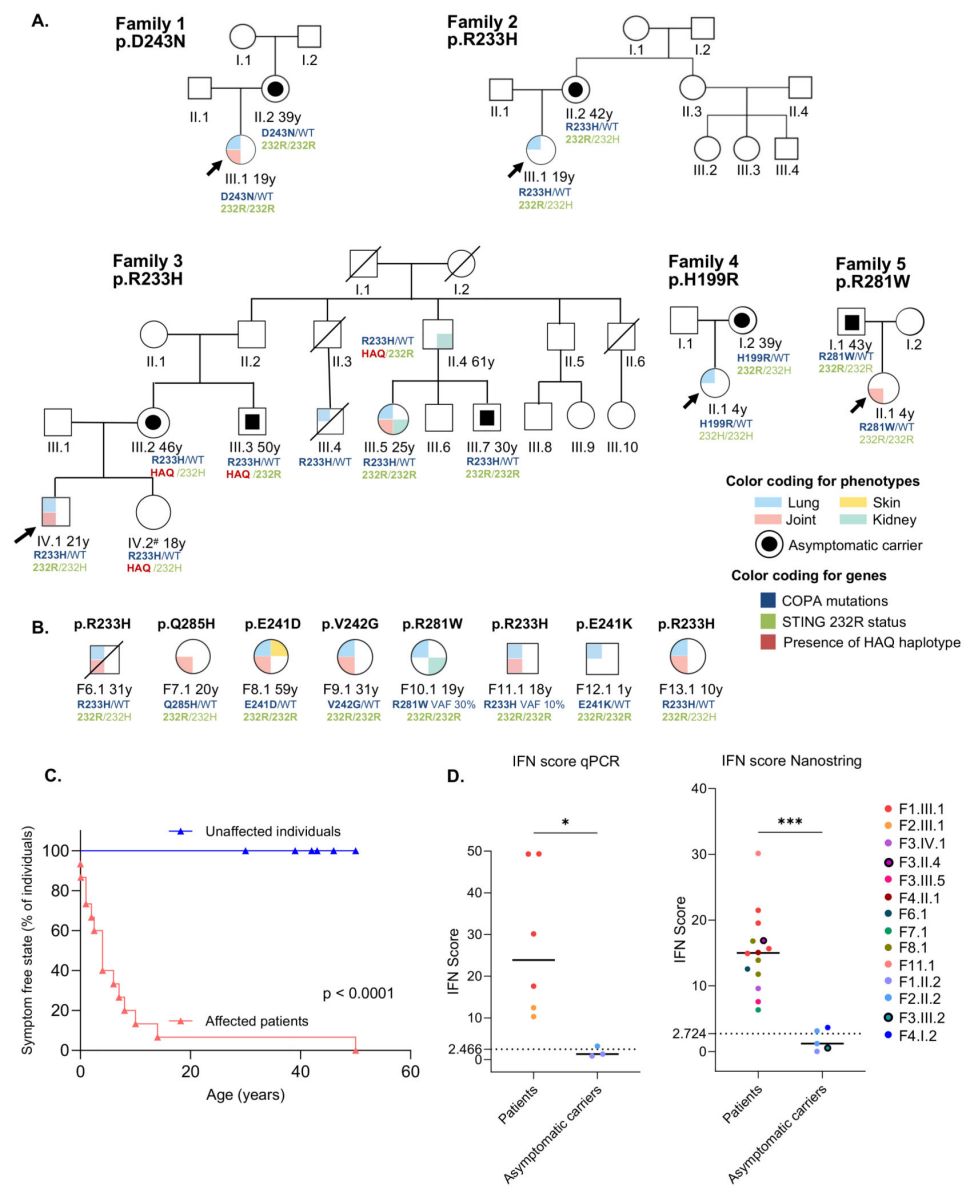
Pedigree structure, genotype and type I interferon signaling status in familial and sporadic cases of COPA syndrome and asymptomatic *COPA* mutation carriers (A) Pedigrees comprising both individuals manifesting COPA syndrome and clinically asymptomatic individuals, and (B) sporadic cases of COPA syndrome in whom STING haplotype data were available. Circles and squares indicate females and males respectively; black fill indicates clinical asymptomatic status. Blue, pink, green and yellow quadrants indicate lung, joint, kidney and skin involvement respectively. COPA genotypes are shown in blue, STING 232 status in green and presence of STING HAQ haplotype in red. Diagonal bars indicate deceased individuals. Arrows indicate index cases. (C) Kaplan–Meier analysis of age at symptom onset in individuals heterozygous for a COPA mutation (log-rank test, p < 0.0001). Affected patients are presented in salmon pink, while clinically asymptomatic individuals are presented in blue. ^#^The status of patient F3.IV.2, manifesting vitiligo in the absence of other disease features at age 18 years, was considered uncertain, so that she was not included in our further analysis (D) Type I interferon pathway activation in individuals harbouring a pathogenic mutation in *COPA* as assessed by interferon (IFN) score performed by qPCR (left panel) or NanoString (right panel). Horizontal bar indicates median. STING HAQ haplotype carriers are circled in black. Mann-Whitney test,*p<0.05, ***p<0.01. Dotted lines indicate the upper control values of 2.466 (qPCR) and 2.724 (NanoString).

We ascertained 23 individuals from 13 European families harbouring an experimentally validated pathogenic mutation in *COPA* (Watkin et al., 2015; Bader-Meunier et al., 2021; Kechiche et al., 2024; David et al., 2025) for whom STING haplotype data from next-generation or Sanger sequencing were also available (Figure 1A and B). Fifteen individuals (65%) were assessed as clinically symptomatic, fourteen of whom manifest classical features of COPA syndrome (lung: 87%, joint: 71%; kidney: 14%) detailed in Table 1 with a median age at onset of 4 years (range 0 – 14 years), and one who was diagnosed with isolated renal disease at age 50 years (see below) (Figure 1C). In contrast, seven individuals (29%) were clinically asymptomatic, with no features of COPA syndrome following clinical assessment (median age 42 years, range 30 - 50 years) (Figure 1C). Clinical non-penetrance in these seven individuals was confirmed by physical evaluation, and in five cases (not including F3.PIII.3 and F3.PIII.7) by investigations including chest computed tomography (CT) scan, pulmonary function assessment and testing for proteinuria. Consistent with these demographic and clinical data, there was a clear distinction in the level of IFN-I signalling recorded in the whole blood between the 15 symptomatic patients versus the seven asymptomatic individuals (Figure 1D). The clinical status of one individual (F3.IV.2), manifesting vitiligo in the absence of other disease features at age 18 years, was considered uncertain, and this HAQ positive individual was not included in our further analysis.

Two asymptomatic individuals ascertained in our cohort, most recently assessed at the ages of 46 and 50 years, were positive for the HAQ haplotype. Notably, however, in contrast to the data of Simchoni et al., five clinically asymptomatic individuals, aged 30, 39, 39, 42 and 43 years at last evaluation, did not carry the HAQ haplotype. Further, the HAQ haplotype was present in a male (F3.II.4) diagnosed with lupus-like membranous glomerulonephritis during evaluation for intra-familial kidney donation to his daughter. Although idiopathic membranous glomerulonephritis can occur in the general population, most typically in males over the age of 60 years, the absence of anti-PLA2R antibodies in both serum and kidney biopsy samples (Beck et al., 2009), along with full-house immunofluorescence, suggests the involvement of the *COPA* mutation in the development of the disease in this individual. Additionally, absence of lung involvement has been previously reported in COPA patients (F7.1 (Bader-Meunier et al., 2021) and F5.II.1 in this cohort), and the IFN score, assessed once in this patient, was elevated to a level comparable with that of other symptomatic patients with a *COPA* mutation (Figure 1D). During his most recent evaluation, at the age of 61 years, while clinically well, the patient remains dependent on hydroxychloroquine and anti-proteinuria treatment to maintain his renal function.

As new genes associated with inborn errors of immunity (IEIs) have been discovered, and the use of high-throughput sequencing techniques has become more widely available, the number of mutant genotypes associated with markedly variable clinical expression and, in some cases, frank clinical non-penetrance, has increased (Gruber and Bogunovic, 2020; Kingdom and Wright, 2022). In the context of the IEIs, well-known examples include genes underlying primary immunodeficiencies (*CTLA4*) and autoinflammation (*IFIH1, RNASEH2B, COPA, JAK1, NLRP3, TNFRSF1A*). Broadly speaking, this phenomenon is most likely explained by additional genetic factors, either protective or susceptibility alleles, as well as epigenetic modifiers and environmental triggers such as infections.

STING signalling is central to host immune defence, but also to other biological processes such as cellular senescence, autophagy and anti-tumor activity, reflecting the fact that STING evolved prior to the development of IFN-I signalling pathways. The STING HAQ haplotype shows a higher prevalence in East Asian populations compared to Europeans and sub-Saharan Africans, suggesting positive selection during migration out of Africa 50,000-70,000 years ago (Yi et al., 2013; Patel et al. 2017). From an immunological standpoint, evaluations of functional differences between STING alleles have been contradictory, with the relative activity of the 232R and HAQ haplotypes highly variable between reports (Koide et al., 2025). *In vitro*, Simchoni et al. showed that HAQ STING acts dominantly to dampen COPA-dependent STING activation, and thus might be protective against the risk of developing COPA syndrome. Their data were remarkable in suggesting a binary distinction between clinical disease and clinical non-penetrance based on HAQ haplotype status, with all nine asymptomatic individuals heterozygous for a *COPA* mutation also being HAQ positive, and an absence of the STING HAQ haplotype in all 26 individuals manifesting clinical disease. In contrast, in our cohort of European patients, we ascertained five clinically asymptomatic individuals harbouring a pathogenic *COPA* mutation in the absence of the STING HAQ haplotype. This observation indicates that clinical penetrance in COPA syndrome involves factors beyond HAQ haplotype status (and which might differ between populations). The identification of an HAQ positive male with isolated kidney disease starting later in life, that we consider most likely attributable to the recurrent R233H mutation in COPA, remains difficult to interpret. This finding raises three possibilities: (1) despite the presence of an HAQ allele, disease expression can occur; (2) the HAQ allele is protective against ‘complete’ disease expression but can sometimes be associated with an ‘attenuated’ phenotype; (3) the HAQ allele is completely protective against disease expression (and this patient’s phenotype is unrelated to his mutation status).

Considering other additive or protective genetic factors explaining penetrance in COPA syndrome, we found the frequency of the 232R allele to be the same in symptomatic and asymptomatic individuals (8/15 (53%) and 4/7 (57%) respectively). We also looked for variants in an in-house panel of approximately 500 other IFN-related genes in whole genome sequencing data available for ten *COPA* mutations carriers, and no potential candidates emerged that were shared between asymptomatic or symptomatic individuals (data not shown). In addition, no obvious environmental or infectious factors were reported in our cohort (although, given the retrospective nature of our data set, additional evaluation of previous viral exposures is warranted). Of note, STING also serves as an innate immune receptor for bacterial cyclic di-nucleotides (Ablasser and Chen, 2019). As such, STING status might modulate the effect of microbiota at (alveolar) epithelial barriers and play a role in the clinical variability seen in STING-mediated inflammation. Further, although not assessed systematically, in the one asymptomatic carrier of a *COPA* mutation tested, we found no neutralizing anti-IFN-I auto-antibodies which might explain an absence of clinical penetrance. Finally, while recent evidence has highlighted monoallelic expression as an explanation for clinical non-penetrance in at least 4% of IEI associated genes, COPA did not show monoallelic expression in a clonally expanded T cell model (Stewart et al., 2025). Other potential explanations for clinical non-penetrance such as somatic mosaicism and mutation reversion have not been reported in COPA syndrome.

Summarizing, our findings challenge the suggestion that STING haplotype status is the sole determinant of clinical penetrance in COPA as highlighted by the identification of five asymptomatic individuals not carrying the HAQ haplotype. While we cannot rule out the possibility of later onset disease in the clinically asymptomatic HAQ negative individuals that we ascertained, disease penetrance has been previously estimated to be 89% by age 12 years, and in the group of affected individuals reported by Simchoni et al. in 2025, 83% of patients were symptomatic by 5 years of age, with 100% manifesting disease by age 18 years (Simchoni et al., 2023; Simchoni et al., 2025). However, given that the number of patients with COPA syndrome reported in the literature remains low, the full spectrum of clinical disease is likely yet undefined, a point possibly illustrated by the HAQ positive male with apparently isolated renal disease that we describe here. Taken together, the recent findings of Simchoni et al. and the results that we present highlight the need to establish international consortia to study the phenomenon of clinical penetrance in COPA syndrome (and other rare autoinflammatory diseases), so as to gather sufficient numbers of patients to define the underlying mechanisms in further clinical and functional studies.

## Materials and methods

### Patients

Patients with genetically confirmed COPA syndrome were included in this retrospective study. Patients were recruited from referent rare diseases centres in France and the United Kingdom. COPA variants were annotated according to HGVS nomenclature using the MANE select transcript NM_004371.4. STING haplotype was determined by next-generation or Sanger sequencing and annotated according to HGVS nomenclature using the MANE select transcript NM_198282.4. Data sharing was consistent with the requirements of the relevant local ethics committee for all patients.

### Data collection

Data collected included sex, clinical manifestations at presentation and during follow-up, and age at genetic diagnosis of COPA syndrome. Results of chest X-ray, chest CT and pulmonary function tests were recorded. ILD was defined according to CT scan findings, and alveolar haemorrhage determined according to CT scan and / or bronchoalveolar lavage and / or lung biopsy. The presence of honeycombing and / or traction bronchiectasis and / or inter and intralobular septal thickening radiologically defined pulmonary fibrosis. Biological parameters such as inflammatory markers, autoantibodies and immunological status were collected, as well as treatment characteristics and the response to therapy.

### IFN pathway assessment

Status of IFN biomarkers was determined by studying the expression of ISGs (by qPCR (Rice et al., 2013) or by NanoString (Kim et al., 2018) in peripheral blood.

### Study approval

The study was approved by the Comité de Protection des Personnes (ID-RCB/EUDRACT: 2014-A01017-40; revalidated in 2022 and 2025). Written informed consent was obtained for all patients.

### Statistics

Analyses were performed using PRISM software (v10, GraphPad Inc.). A *p*-value less than 0.05 was considered significant.

## Figures and Tables

**Table 1:** Clinical and genetic characteristics of the individuals in our cohort

Patient	Gender	Ethnic background	COPA mutation	STING haplotype status	Age at last evaluation (years)	Age at onset	Clinical presentation	Kidney function	Chest CT /CXR (age)	PFT (age)	Auto Abs*	IFN score (age)	Chronic immunosuppressive treatment
*F1.PII.2*	*F*	*Caucasian*	*D243N*	*Non-HAQ*	*39*	-	*Asymptomatic*	*Normal*	*Normal CXR* *(36)*	*NA*	*Negative*	*Negative* *(30)*	*None*
F1.PIII.1	F	Caucasian	D243N	Non-HAQ	19	2.5	ILD and arthralgias	Normal	Cystic lung disease	Lung restriction	ANA, RF	Positive	Steroids, MMF, rituximab, bari
*F2.PII.2*	*F*	*Caucasian*	*R233H*	*Non-HAQ*	*42*	-	*Asymptomatic*	*Normal*	*Normal CT* *(35)*	*Normal* *(35)*	*Negative*	*Minimally positive (35)*	*None*
F2.PIII.1	F	Caucasian	R233H	Non-HAQ	19	2	Recurrent AH	Normal	Ground glass opacities and cysts	Lung restriction	ANA, RF	Positive	Cyclophosphamide, MMF, AZA, anti- IL1, ruxo
F3.PII.4	M	Caucasian	R233H	HAQ	61	50	Membranous glomerulonephritis	Abnorma l	Normal	Normal	NA**	Positive	HCQ
*F3.PIII.2*	*F*	*Caucasian*	*R233H*	*HAQ*	*46*	-	*Asymptomatic*	*Normal*	*Normal CT* *(39)*	*Normal* *(39)*	*Negative*	*Negative* *(39)*	*None*
*F3.PIII.3*	*M*	*Caucasian*	*R233H*	*HAQ*	*50*	-	*Asymptomatic*	*NA*	*NA*	*NA*	*NA*	*NA*	*None*
F3.PIII.5	F	Caucasian	R233H	Non-HAQ	25	10	AH, arthritis and glomerulonephritis	Kidney Tx	Pulmonary hemosideros is	Normal	Negative	Positive	Steroids, cyclophosphamide, MMF, Kidney Tx
*F3.PIII.7*	*M*	*Caucasian*	*R233H*	*Non-HAQ*	*30*	-	*Asymptomatic*	*NA*	*NA*	*NA*	*NA*	*NA*	*None*
F3.PIV.1	M	Caucasian	R233H	Non-HAQ	21	1	AH and arthralgias	Normal	Alveolar condensation	NA	ANA, MPO	Positive	Steroids, MMF, AZA, rituximab, ruxo
F3.PIV.2#	F	Caucasian	R233H	HAQ	18	-	Vitiligo	Normal	Normal CT (2018)	Normal (2018)	*Negative*	NA	None
*F4.PI.2*	*F*	*Caucasian*	*H199R*	*Non-HAQ*	*39*	-	*Asymptomatic*	*Normal*	*Normal CT* *(37)*	*Normal* *(37)*	NA	*Minimally positive (36)*	*None*
F4.PII.1	F	Caucasian	H199R	Non-HAQ	4	0	Prematurity, interstitial lung disease	Normal	Ground glass opacities and cysts	NA	*NA*	Positive	Steroids, ruxo
*F5.PI.1*	*M*	*Caucasian*	*R281W*	*Non-HAQ*	*43*	-	*Asymptomatic*	*Normal*	*Normal CT* *(43)*	*Normal* *(43)*	ANA, anti- CCP, RF	*NA*	*None*
F5.PII.1	F	Caucasian	R281W	Non-HAQ	6.5	4	Polyarthritis	Normal	Normal	NA	ANA, anti- RNP, ANCA	Positive	MTX, adalimumab
F6.P1	M	North African	R233H	Non-HAQ	31 (deceased)	7	ILD and polyarthritis	Normal	Honeycombi ng, ground glass opacities	Lung obstruction and restriction	RF, anti- CCP	Positive	Steroids, HCQ, lung Tx
F7.P1	F	Caucasian	Q285H	Non-HAQ	20	6	Polyarthritis	Normal	Normal	Normal	ANA, MPO, anti- RNP	Positive	MTX, rituximab
F8.P1	F	Caucasian	E241D	Non-HAQ	59	14	ILD, polyarthritis and chilblains	Normal	Honeycombi ng, ground glass opacities	Lung obstruction and restriction	ANA, MPO	Positive	MTX, leflunomide, AZA, rituximab, tocilizumab, MMF, HCQ, bari
F9.P1	F	North African	V242G	Non-HAQ	31	8	ILD and polyarthritis	Normal	Cysts, ground glass opacities	Lung obstruction and restriction	ANA, MPO	Positive	Steroids, MTX, AZA, Tocilizumab, infliximab, etanercept, filgotinib
F10.P1	F	NA	R8281W, mosaic	Non-HAQ	19	4	Glomerulonephrit is and ILD	Glomerul onephritis	Ground glass opacities	Isolated decrease of DLCO	RF, anti- CCP	Positive	Steroids, AZA, rituximab
F11.P1	M	NA	R233H, mosaic	Non-HAQ	18	4	ILD and polyarthritis	Normal	Cysts, ground glass opacities	Lung obstruction and restriction	MPO	Positive	MTX, adalimumab, bari
F12.P1	M	NA	E241K	Non-HAQ	1	0.1	ILD	Normal	Ground glass opacities	NA	ANA	Positive	Bari
F13.P1	F	North African	R233H	Non-HAQ	10	1	ILD and arthralgias	Normal	Honeycombi ng, cysts	Lung restriction		Positive	HCQ, ruxo

Abbreviations: AH: alveolar haemorrhage; ANA: antinuclear antibodies; AZA: azathioprine; Bari: baricitinib; CCP: cyclic citrullinated peptide; CT: computed tomography; HCQ: hydroxychloroquine; IL1: interleukin-1; ILD: interstitial lung disease; MMF: mycophenolate mofetil; MPO: myeloperoxidase; MTX: methotrexate; NA: not assessed; RF: rheumatoid factor; RNP: ribonucleoprotein; Ruxo: ruxolitinib; Tx: transplantation; WT: wild type.

# The disease status of F3.IV.2, manifesting vitiligo in the absence of other disease features at age 18 years, was considered uncertain and was not included in our further analysis.

* AutoAbs refer to ANA, RF, anti-CCP and ANCA (Anti-neutrophil cytoplasmic antibody).

** F3.PII.4 was only tested for anti-PLA2R antibodies that were negative.

Asymptomatic patients are described in italicized font.

## Data Availability

Data underlying Figure 1 are available in the published article. In order to maintain confidentiality, sequence data relating to individual patients are not available.

## Summary

It has been suggested that the presence of the HAQ STING allele explains the high rate of clinical non-penetrance observed among individuals carrying a pathogenic mutation in COPA. David et al. identify multiple asymptomatic individuals lacking the HAQ allele, indicating that STING haplotype status alone does not account for disease expression in COPA syndrome.
